# Gas-permeable ethylene bags for the small scale cultivation of highly pathogenic avian influenza H5N1 and other viruses in embryonated chicken eggs

**DOI:** 10.1186/1743-422X-7-23

**Published:** 2010-01-28

**Authors:** Sara B Hamilton, Deirdre E Daniels, William A Sosna, Eric R Jeppesen, Julie M Owells, Micah D Halpern, Kimberly S McCurdy, Jonathan O Rayner, John A Lednicky

**Affiliations:** 1 Energy and Life Sciences Division, Midwest Research Institute, 425 Volker Boulevard, Kansas City, Missouri, 64110, USA; 2 Biosafety/Biosurety Office, Midwest Research Institute, 425 Volker Boulevard, Kansas City, Missouri, 64110, USA

## Abstract

**Background:**

Embryonated chicken eggs (ECE) are sometimes used for the primary isolation or passage of influenza viruses, other viruses, and certain bacteria. For small-scale experiments with pathogens that must be studied in biosafety level three (BSL3) facilities, inoculated ECE are sometimes manipulated and maintained in small egg incubators within a biosafety cabinet (BSC). To simplify the clean up and decontamination of an egg incubator in case of egg breakage, we explored whether ethylene breather bags could be used to encase ECE inoculated with pathogens. This concept was tested by determining embryo survival and examining virus yields in bagged ECE.

**Results:**

Virus yields acceptable for many applications were attained when influenza-, alpha-, flavi-, canine distemper-, and mousepox viruses were propagated in ECE sealed within ethylene breather bags.

**Conclusions:**

For many small-scale applications, ethylene breather bags can be used to encase ECE inoculated with various viruses.

## Background

Embryonated (embryonating) chicken eggs (ECE) have long been used for isolating or propagating influenza and other viruses and certain bacteria such as *Rickettsia *[1-5]. Alpha-, corona-, flavi-, paramyxo-, and poxviruses are among the non-influenza viruses sometimes grown in ECE. For small-scale work with pathogens that must be worked with in BSL3 facilities, inoculated ECE are sometimes housed in small egg incubators kept within a BSC [such a practice is not practical for medium-to-large diagnostic operations, wherein ECE are placed in incubators within a bioBubble (Ft. Collins, CO) or similar barrier and containment enclosure]. Since ECE are fragile, accidental egg breakage is possible. Furthermore, diagnostic specimens inoculated into ECE may contain contaminating flora that form enough gas to break the egg shell. We sought a simple method to contain spillage from a broken ECE inoculated with dangerous pathogens, and explored the feasibility of using ethylene breather bags for that purpose. Ethylene breather bags are permeable to oxygen and carbon dioxide but retain water, and are used in the aquarium industry to transport live fish. Chicken embryo survival was examined and the yield of various influenza and other viruses in bagged eggs was determined.

## Results

### 1. Embryo survival

No differences were detected in the survival of chicken embryos in bagged vs non-bagged 7 - 12 day old ECE after five days of incubation without rotation as performed for virus-inoculated ECE. Noteworthy, especially during summer months, up to 20% attrition (death of non-inoculated ECE) occurred with some batches, regardless of whether the ECE were bagged or not bagged. Since the ECE are checked and culled if dead upon receipt from the supplier and again immediately prior to use, the deaths have been attributed to failure to thrive under normal circumstances. Since the ECE are not rotated, a factor contributing to attrition may be attachment of the embryo to the egg-shell and its subsequent deleterious deformation/improper development.

### 2. Propagation of Influenza viruses in bagged ECE

**Figure 1:**
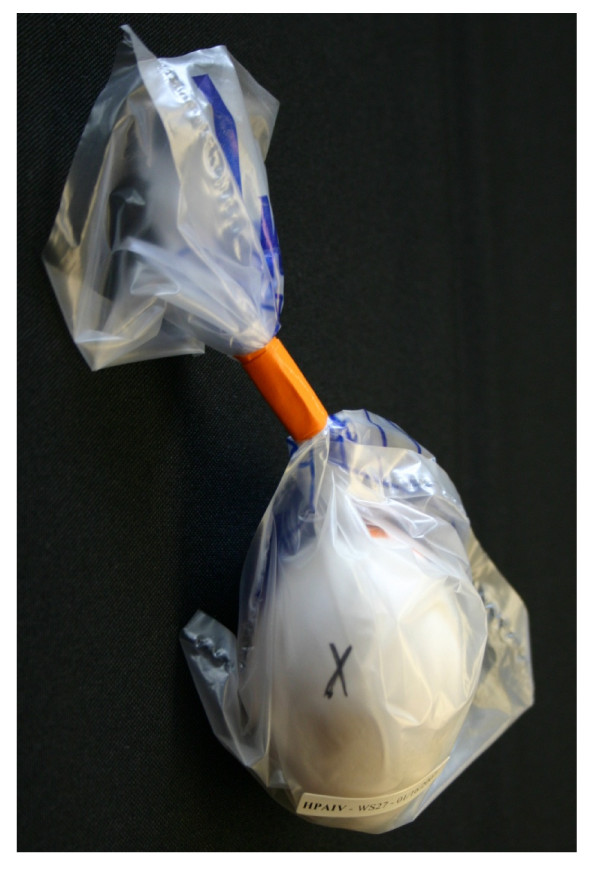
**Virus-inoculated ECE enclosed in an ethylene breather bag**. The embryo's position prior to inoculation of the ECE with virus was marked with the letter "X".

Various type A and B influenza viruses were grown to levels acceptable for our applications in ECE in ethylene breather bags. It was not necessary to add water to humidify the interiors of sealed bags. Compared to bags containing eggs without extraneously added moisture, virus yields and embryo development were similar when up to one ml of sterile water or a moistened filter were placed with eggs in bags (data not shown). Virus growth occurred regardless of the inoculation route/site and storage orientation (prone or horizontal) of the egg (data not shown). An example of a virus-inoculated egg in a breather bag is shown in Figure 1. Comparisons of virus titers calculated as 50% tissue culture infectious dose (TCID_50_) in Madin-Darby canine kidney (MDCK) cells and 50% egg infectious dose (EID_50_) in ECE of two influenza viruses strains grown in the chorioallantoic sac (CAS) of ECE (incubated prone, with air sac atop) are given in Table 1. Representative titers (TCID_50/_ml) obtained for various other influenza A and B viruses are given in Tables 2, 3, 4, 5, 6, and 7. As previously observed, some recent influenza virus H3N2 isolates from humans, such as A/Brisbane/10/2007 (H3N2) [Table 3] produced low virus titers during primary passage in ECE [6,7].

**Table 1:** Yields Obtained for *Influenza Virus *Grown in Bagged^a ^vs Non-bagged ECE^a^.

	Bagged ECE		Non-bagged ECE	
Virus Strain	**TCID**_**50**_^**b**^	**EID**^**c**^	**TCID**_**50**_	EID
A/NWS/1933 (H1N1)	8.05 ± 0.15	9.25 ± 0.25	8.1 ± 1.0	9.4 ± 0.2

A/HK/8/1968 (H3N2)	7.95 ± 0.05	9.3 ± 1.0	7.75 ± 0.25	9.0 ± 0.2

^a^CAS fluids from four ECE were harvested and pooled 48 hrs after infection of 9-day old ECE with 10^2 ^TCID_50 _units of virus and incubation at 34°C. The experiment was repeated three separate times. ^b^Log_10 _of TCID_50_/ml in MDCK cells in serum-free growth medium plus trypsin. ^c^Log_10 _of EID_50_/ml obtained in non-bagged 10-day-old ECE incubated at 34°C.

**Table 2:** Yields Obtained for *Influenza virus *H1N1 Strains Grown in Bagged ECE.

Strain	Specimen source	**Log**_**10**_**TCID**_**50**_**/ml**^**a**^
A/Puerto Rico/8/1934	Human	7.5

A/New Caledonia/20/1999	Human	7.9

A/Hawaii/15/2001	Human	7.9

A/Solomon Islands/03/2006	Human	7.0

A/New York/18/2009	Human	8.0

A/Mexico/408/2009	Human	7.0

^a^TCID_50_/ml of CAS fluids in MDCK cells in serum-free growth medium plus trypsin. CAS fluids from four ECE were harvested and pooled 48 hrs after infection of 9-day old ECE with 10^2 ^TCID_50 _units of virus and incubation at 34°C

**Table 3:** Yields Obtained for *Influenza virus *H3N2 Strains Grown in Bagged ECE.

Strain	Specimen source	**Log**_**10**_**TCID**_**50**_**/ml**^**a**^
A/New York/55/2004	Human	7.9

A/Wisconsin/67/2005	Human	8.0

A/Hiroshima/52/2005	Human	6.9

A/Brisbane/10/2007	Human	5.0

^a^TCID_50_/ml of CAS fluids in MDCK cells in serum-free growth medium plus trypsin. CAS fluids from four ECE were harvested and pooled 48 hrs after infection of 9-day old ECE with 10^2 ^to 10^3 ^TCID_50 _units of virus and incubation at 34°C.

**Table 4:** Yields Obtained for Miscellaneous *Influenza virus *Type A Strains Grown in Bagged ECE

Strain	Specimen source	**Log**_**10**_**TCID**_**50**_**/ml**^**a**^
A/Alberta/79/2003 (H2N3)	Mallard	6.0

A/Wisconsin/1968 (H5N9)	Turkey	7.9

A/New York/107/2003 (H7N2)	Human	5.6

A/Netherlands/219/2003 (H7N7)	Human	9.7

A/Hong Kong/G9/1997 (H9N2)	Chicken	8.2

^a^TCID_50_/ml of CAS fluids in MDCK cells in serum-free growth medium plus trypsin. CAS fluids from four ECE were harvested and pooled 48 hrs after infection of 9-day old ECE with 10^2 ^to 10^3 ^TCID_50 _units of virus and incubation at 34°C.

**Table 5:** Yields Obtained with H5N1 Reverse Genetics Constructs in an A/PR/8/1934 Vaccine Strain Background Grown in Bagged ECE.

Strain	**Log**_**10**_**TCID**_**50**_**/ml**^**a**^
A/Anhui/01/2005 (H5N1)-PR8-IBCDC-RG	8.7

A/VNH5N1-PR8/CDC-RG	10.0

^a^TCID_50_/ml of CAS fluids in MDCK cells in serum-free growth medium plus trypsin. CAS fluids were pooled from four ECE and harvested 48 - 72 hrs after infection of 9-day old ECE with 10^2 ^to 10^3 ^TCID_50 _units of virus and incubation at 34°C.

**Table 6:** Yields Obtained with *Influenza virus *H5N1Strains Grown in Bagged ECE.

Strain	Specimen source	**Log**_**10**_**TCID**_**50**_**/ml**^**a**^
A/Hong Kong/220/97	Chicken	9.0

A/Hong Kong/156/97	Human	9.4

A/Hunan/795/2002	Duck	9.0

A/Yunnan/1251/2003	Chicken	7.0

A/Vietnam/1203/2004	Human	8.3

A/Mongolia/244/2005	Whooper Swan	8.0

A/Iraq/207-NAMRU3/2006	Human	10.0

A/Hong Kong/45/2006	Scaly Breasted Munia	8.0

A/Hong Kong/645/2006	Common Magpie	10.0

A/Hong Kong/1038/2006	Japanese White Eye	10.0

A/Hong Kong/D-06-0947/2006	Chicken	8.0

A/Korea/IS/2006	Chicken	9.0

^a^TCID_50_/ml of CAS fluids in MDCK cells in serum-free growth medium plus trypsin. CAS fluids were pooled from four ECE and harvested 48 - 72 hrs after infection of 9-day old ECE with 10^2 ^to 10^3 ^TCID_50 _units of virus and incubation at 37°C.

**Table 7:** Yields Obtained for *Influenza virus *B Strains Grown in Bagged ECE.

Strain	**Log**_**10**_**TCID**_**50**_**/ml**^**a**^
B/Ohio/01/2005 (Victoria/2/87-like)	9.0

B/Florida/07/2004 (Yamagata/16/88-like)	8.0

B/Malaysia/2506/2004	7.0

B/Florida/04/2006	7.1

^a^TCID_50_/ml of CAS fluids in MDCK cells in serum-free growth medium plus trypsin. CAS fluids were pooled from four ECE and harvested 72 hrs after infection of 9-day old ECE with 10^2 ^to 10^3 ^TCID_50 _units of virus and incubation at 34°C.

### 3. Canine distemper virus

Egg-adapted *Canine distemper virus *(CDV) strain Lederle (American-1 lineage) obtained from the American Type Culture Collection (ATCC, Manassas, VA) grew readily in bagged ECE, evidenced by RT-PCR detection of CDV RNA in isolated chorioallantoic membrane (CAM) five days post-infection (p.i.). Changes in the general appearance (of the CAM) were also visible without staining and microscopic evaluation of isolated CAM. In contrast, wild-type CDVs from canine specimens required two to three serial passages before facile detection (by RT-PCR or visually apparent changes in the appearance of the CAM). Titers of 10^6 ^- 10^7 ^pfu/ml were obtained from isolated CAM with CDV-Lederle upon first passage with a starting inoculum of 10^2 ^pfu/ECE in both bagged and non-bagged ECE, whereas 10^1 ^- 10^2 ^pfu/ml were obtained with wild-type American-2 lineage CDVs (titer of CDV in initial inoculum unknown) in bagged and non-bagged ECE. Similarly, after three serial passages of wild-type CDVs with a starting inoculum of 10^2 ^pfu/ECE in either bagged or non-bagged ECE, the yields of cell-associated virus (CAM-associated virus) were around 10^3 ^pfu/ml, and were not detectable to 10^1 ^pfu/ml for CDV isolates in allantoic fluid (details to be presented elsewhere).

### 4. *Mousepox virus *strain Moscow

"Pocks" were visible on the CAM of bagged ECE at 3 days post-infection (data not shown).

### 5. *Venezuelan equine encephalitis virus *strain Trinidad donkey ((VEEV-Td)

Chicken embryos were usually killed (by the virus) within 24 hrs after infection with 10^3 ^pfu of VEEV-Td per ECE. Virus yields in bagged ECE were: CAM > brain (head) > body > allantoic fluid > yolk sac (YS) membrane. Virus yields from homogenized CAM were generally around 3 × 10^8 ^pfu/mL whereas homogenized brain tissue yielded about 4 × 10^7 ^pfu/mL as measured by plaque assays in African green monkey kidney (Vero) cells. Though a direct comparison with VEEV-Td grown in non-bagged ECE was not allowed due to biosafety rules imposed by our institute, the virus yields from the CAM are within the expected range for alphaviruses grown in ECE based on historic data [8].

### 6. *Japanese encephalitis virus *strain Nakayama (JEV-Nak)

Chicken embryos usually died between 48 - 72 hrs after infection with 10^3 ^pfu of JEV-Nak per ECE. Virus yields in bagged ECE were: brain (head) > body > allantoic fluid > CAM > YS membrane. Virus yields from homogenized brain tissue were generally around 4 × 10^5 ^pfu/mL whereas homogenized body tissue yielded about 4 × 10^4 ^pfu/mL by plaque assay in Vero cells. A direct comparison of our stock of JEV-Nak grown in non-bagged ECE was not allowed due to biosafety rules imposed by our institute.

## Discussion

Various viruses were successfully cultivated in ECE in ethylene breather bags. No defective bags (defined as bags with obvious holes) were observed during this work. Whereas additional safety is inferred since the gas-permeable ethylene bags retain water (and thus much larger virus particles should also be retained), this has not been extensively tested at MRI. However, a preliminary test supports this thesis: T4 phage inside intact bags did not infect *E. coli *when the bags were immersed in flasks containing the bacteria for 24 hrs (starting out with log-phase bacteria). In contrast, the phage infected *E. coli *when pin-holes were made in the bags prior to immersion in flasks containing log-phase bacterial cultures. Though exhaustive tests were not performed, chicken embryos past seven days of development did not thrive in various other types of plastic bags.

Influenza virus yields in bagged ECE were consistent with expectations; for example, titers within the range of 10^6 ^to 10^9 ^pfu/ml (infectivity measured in MDCK cells) are common for primary or low-passage virus isolates provided contaminating microbial flora normally present in clinical specimens are suppressed by antibiotic treatment. The variability in virus yield is also dependent on viral strain. The growth and yield of other viruses (CDV, *Mousepox virus*, JEV-Nak, and VEEV-Td) in bagged ECE was also consistent with expectations. Whereas some JEV strains that have been adapted for growth in ECE attain higher viral yields than was attained here, it is not considered aberrant to attain the lower yields with fresh or low-passage JEV stocks. Similarly, wild type CDVs may require adaptation and serial passage in ECE before vigorous growth is detected [9,10], as observed here.

For small-scale operations using relatively few ECE for the propagation of BSL3 pathogens, breather bags are convenient for containing spillage from broken eggs. Use of the bags for the application described here offers one solution over the need to purchase or construct additional barrier and containment enclosures. There are two drawbacks: bagging ECE imposes additional labour constraints, and candling of the eggs through the bags can be challenging especially when numerous eggs are present in a bag. However, this technique is only intended for small-scale experiments requiring few ECE. The same idea may be useful for tissue culture plate systems since they have the same problem if dropped; i.e. as an added safety feature.

For larger-scale needs, other primary containment devices could achieve the same goal of increased containment such as tradition egg incubators placed inside a primary containment device such a BSC or high-efficiency particulate arrestor (HEPA) - filtered vinyl enclosures. One alternative would be the use of an egg incubator with removable self-sealing modular containers for eggs. A large stand alone incubator could thus be positioned somewhere outside the BSC, and inoculated ECE safely transported in and out of the BSC and the containers with ECE repositioned into designated racks in the incubator. Midwest Research Institute is currently building such a device.

## Conclusions

For small-scale operations, ethylene breather bags can be used to enclose ECE infected with influenza viruses, CDV, *Mousepox virus*, JEV-Nak, and VEEV-Td as a precaution to contain possible spillage from broken eggs. We predict these bags will work for ECE infected with other viruses as well, and may be useful for tissue culture plate systems as well. Intended for small scale projects, the procedure is not practical for medium- to large-scale operations.

## Methods

### 1. Breather bags

Ethylene breather bags were obtained from Kordon LLC (Hayword, CA). For up to three ECE, 5.5 × 8 inch bags were used, whereas 7.5 × 12 inch bags were used for small-batch cultivation of up to six ECE inoculated with virus.

### 2. Embryonated chicken eggs

Specific pathogen-free (SPF) *Chicken anemia virus *(CAV)-free ECE were obtained from Charles River Laboratories **(**Wilmington, MA).

### 3. Influenza virus propagation in embryonated chicken eggs

Various methods for the growth of influenza viruses in ECE were tested. The primary method was by inoculation of the CAS. Amniotic sac (AS) inoculation (solely or in combination with CAS inoculation), YS, and CAM routes of inoculation were also tested for some virus strains. Inoculation into the AS is technically demanding and primarily reserved for isolating influenza viruses from humans when standard methods are shown to be insufficient or for high-priority efforts. Inoculations into the YS and CAM are performed for the primary isolation of some avian influenza viruses from non-chicken species and some swine influenza viruses [[11], and J. Lednicky, unpublished observations]. For CAS, AS, and CAM inoculations, 9 to 11 day-old ECE were used (for AS inoculations, ECE up to 14 days old were also tested). ECE that were 7 - 9 days-old were used for YS inoculation. For CAS and top-side AS inoculations, the embryo was located by candling, the egg-top decontaminated with alcohol, and a hole punched over the air sac without piercing the CAM. The ECE were inoculated with up to 100 μl of virus-containing material using a 1 ml tuberculin syringe and 22-gauge, 1.5-inch (~4 cm) needle, the hole sealed with laboratory tape, and the egg incubated at 34°C for most influenza A or B strains, and at 37°C for H5N1 strains. During inoculation, efforts are made to avoid damage of the CAM, which can result in hemorrhage that leads to death of the embryo.

Top- side inoculation of the AS was performed by: (a) direct inoculation while candling to guide the needle beside the embryo, or (b) by sending the needle through the natural air sac until the needle touched the embryo; upon contact with the embryo, the needle's opening was in the AS. Sideway inoculation of the AS was also evaluated after first creating a false air-space beside the embryo. Similar manipulations were used for YS and CAM inoculations. The incubation period varied according to virus type and strain; in general, up to 24 hrs were used for highly pathogenic H5N1 viruses, 48 - 72 hrs (or longer) for seasonal and type B influenza viruses.

Post-inoculation, the eggs were placed in ethylene bags, the top part of the bag rolled over individual or multiple eggs, and the rolled plastic secured using a rubber band, paper clamp, or laboratory tape. Attempts were made to collect virus just before death of the embryo. Prior to harvest, the eggs were refrigerated for up to 18 hrs to kill the embryo and constrict the blood vessels (and thus reduce bleeding into the AS during harvest). Virus was then harvested as appropriate to the inoculation site and aliquots of the virus stored at -80°C for up to one year or in liquid nitrogen for long-term (>one year) storage. Influenza virus genomic sequences were analyzed by isolating viral RNAs from allantoic fluid (QIAamp Viral RNA kit; QIAGEN, Valencia, CA) and performing two-step reverse transcription-PCR with synthetic universal and other oligonucleotide primers [12,13]. The sequences were determined using an Applied Biosystem 3130 DNA analyzer, BigDye Terminator (v. 3.1) chemistry, and the same oligonucleotide primers used for RT-PCR. Specific details on the primers used for influenza A and B viruses are available upon request.

### 4. Canine distemper virus isolation and propagation of egg-adapted CDV strains in ECE

Egg-adapted CDV-Lederle was purchased from the ATCC. Six to eight day old ECE were used, with inoculation to the CAM. The inoculated eggs were observed daily to monitor embryo viability, and chilled to 4°C after 5 days' incubation and the CAMs harvested and homogenized to a 10% w/v suspension in phosphate-buffered saline with 0.5% w/v purified BSA fraction V. The homogenate was clarified with a low speed spin for 10 min at 4°C, and the supernatant used either as an inoculum for the succeeding passage or stored at -80°C. Wild-type CDVs were isolated from lung and/or brain homogenates of dogs with distemper (data to be presented elsewhere). CDV isolates were analyzed by RT-PCR and nucleotide sequence analyses as previously described [14,15], and viral titers calculated as plaque forming units/ml in Vero cells expressing engineered canine signalling lymphocyte activating molecule (cSLAM) 5 days p.i. (details to be presented elsewhere).

### 5. *Mousepox virus *propagation in ECE

Suspensions of *Mousepox virus *in PBS (0.1 - 0.5 ml) were inoculated onto the CAM of 10 - 12 day old ECE. The ECE were incubated for 3 days at 37°C; virus growth was evidenced by the presence of "pocks" on the CAM [16]. Viral titers (TCID_50_) were determined in BSC-1 cells.

### 6. VEEV propagation in ECE

VEEV was inoculated into the YS of 6 - 8 day old ECE, and incubated at 35 -37°C for up to 24 hrs. The propagated virus was analyzed by plaque assay and full-genomic sequencing (details to be presented elsewhere).

### 7. JEV propagation in ECE

JEV was inoculated into the YS of 8 - 9 day old ECE, and incubated at 35 -37°C for 48 - 72 hrs. The propagated virus was analyzed by plaque assay and full-genomic sequencing (details to be presented elsewhere).

### 8. Tissue culture cells

MDCK and Vero cell lines were obtained from the ATCC, or from Diagnostic Hybrids, Inc. (Athens, OH). The cells were propagated in Dulbecco's Modified Eagle's Medium (DMEM) supplemented with L-Alanyl-L-Glutamine (GlutaMAX™, Invitrogen Corp., Carlsbad, CA), antibiotics [PSN: penicillin, streptomycin, neomycin (Invitrogen Corp.], bicarbonate, and gamma-irradiated heat inactivated fetal bovine serum (HyClone, Thermo Fisher Scientific, Inc., Pittsburgh, PA). The MDCK and Vero cells tested negative for mycoplasma DNA using a Takara PCR Mycoplasma Detection kit (Takara Bio, USA, Thermo Fisher).

### 9. Biocontainment facilities and additional safety precautions

*In-vitro *experiments with H5N1 viruses, JEV, and VEEV, and their cultivation in ECE were conducted in an USDA-approved BSL3-enhanced (BSL3+) containment facility.

### 10. Propagation of Influenza viruses in MDCK cells

Viruses were grown in MDCK cells in serum-free DMEM media supplemented with bicarbonate, antibiotics, and 1.0 μg/mL L-1-tosylamido-2-phenylethyl chloromethyl ketone (TPCK)- treated, mycoplasma- and extraneous virus-free trypsin (Worthington Biochemical Company, Lakewood, NJ) at 34 - 37°C (as appropriate for each virus strain) in 5% CO_2_.

### 11. Determination of TCID_50 _values

TCID_50 _values were calculated for influenza viruses and *Mousepox virus *by the Reed-Muench method [17]. For these determinations, influenza viruses were incubated for 5 days in MDCK, and *Mousepox virus *for 4 days in BSC-1 cells.

### 12. Plaque assays

Standard plaque assays using agarose overlays were used to determine JEV and VEEV titers in Vero cells [18].

## Competing interests

The authors declare that they have no competing interests.

## Authors' contributions

SBH grew influenza viruses, JEV, and VEEV, interpreted data, helped train technicians, and helped draft the manuscript; DED grew influenza viruses, interpreted data, oversaw the training of technicians, and managed the influenza virus programs; WAS grew JEV, VEEV, and influenza viruses, and interpreted data; ERJ is the MRI biosafety officer and recommended evaluation of methodologies that might reduce biohazards stemming from broken virus-inoculated ECE; JMO grew JEV and VEEV; KSM grew JEV and VEEV and managed alpha- and flavivirus programs; MDH performed molecular genetic studies including sequence analyses and alignments; JOR performed JEV and VEEV studies; JAL conceived of using ethylene breather bags for this application, isolated CDV, participated in molecular genetic studies and sequence analyses, interpreted data, oversaw the training of technicians, and drafted the manuscript. All authors read and approved the final manuscript.

## References

[B1] Woodruff AM Goodpasture EW The susceptibility of the chorio-allantoic membrane of chick embryos to infection with the fowl-pox virus Am J Path 1931 7 209 222.5 19969963 PMC2062632

[B2] Burnet F Influenza virus infections of the chick embryo by the amniotic route Aust J Exp Biol Med Sci 1940 18 353 360 10.1038/icb.1940.32

[B3] Burnet FM Growth of influenza virus in the allantoic cavity of the chick embryo Aust J Exp Biol Med Sci 1941 19 291 295 10.1038/icb.1941.44

[B4] Henle W Henle G Stokes JJ Demonstration of the efficacy of vaccination against influenza type A by experimental infection of human beings J Immunol 1943 46 163 175

[B5] Beveridge WIB Burnet FM The cultivation of viruses and rickettsiae in the chick embryo Med Res Council 1946 Special Report No. 256

[B6] Lu B Zhou H Ye D Kemble G Jin H Single amino acid substitutions in the hemagglutinin of influenza A/Singapore/21/04 (H3N2) increase virus growth in embryonated chicken eggs Vaccine 2006 24 44 46 10.1016/j.vaccine.2005.01.116 16814431

[B7] Lu B Zhou H Chan W Kemble G Jin H Improvement of Influenza A/Fujian/411/02 (H3N2) virus growth in embryonated chicken eggs by balancing the hemagglutinin and neuraminidase activities using reverse genetics J Virol 2005 79 6763 6771 10.1128/JVI.79.11.6763-6771.2005 15890915 PMC1112156

[B8] Bang FB The course of experimental infection of the chick embryo with the virus of equine encephalomyelitis J Exp Med 1943 77 337 344 10.1084/jem.77.4.337 19871287 PMC2135341

[B9] Appel MJG Gillespie JH Canine distemper virus Virol Monog 1972 11 1 96

[B10] Schönbauer M Kölbl S Schönbauer-Längle A Perinatale staupeinfektion bei drei eisebären (*Ursus maritimus*) und bei einem brillenbären (*Tremarctos ornatus*) Verh Int Symp Erkrank Zoot 1984 26 131 136

[B11] Woolcock PR McFarland MD Lai S Chin RP Enhanced recovery of avian influenza viruses by a combination of chicken embryo inoculation methods Avian Dis 45 1030 1035 10.2307/1592884 11785874

[B12] World Health Organization Manual on Animal Influenza Diagnosis and Surveillance http://www.who.int/vaccine_research/diseases/influenza/WHO_manual_on_animal-diagnosis_and_surveillance_2002_5.pdf

[B13] Hoffmann E Stech J Guan Y Webster RG Perez DR Universal primer set for the full-length amplification of all influenza A viruses Arch Virol 2001 146 2275 2289 10.1007/s007050170002 11811679

[B14] Lednicky JA Dubach J Kinsel MJ Meehan TP Bocchetta M Hungerford LL Sarich NA Witecki KE Braid MD Pedrak C Houde CM Genetically distant American *Canine distemper virus *lineages have recently caused epizootics with somewhat different characteristics in raccoons living around a large suburban zoo in the USA Virol J 2004 1 e2 10.1186/1743-422X-1-2 PMC524033 15507154

[B15] Lednicky JA Meehan TP Kinsel MJ Dubach J Hungerford LL Sarich NA Witecki KE Braid MD Pedrak C Houde CM Effective primary isolation of wild-type canine distemper virus in MDCK, MV1 Lu and Vero cells without nucleotide sequence changes within the entire haemagglutinin protein gene and in subgenomic sections of the fusion and phospho protein genes J Virol Methods 2004 118 147 157 10.1016/j.jviromet.2004.02.004 15081610

[B16] Fenner F Foster H, Small JD, Fox JG Mousepox The mouse in biomedical research 1982 II Academic Press 209 230

[B17] Reed LJ Muench H A simple method for estimating fifty percent endpoints Am J Hyg 1938 27 493 497

[B18] Powers AM Brault AC Shirako Y Strauss EG Kang W Strauss JH Weaver SC Evolutionary relationships and systematics of the alphaviruses J Virol 2001 75 10118 10131 10.1128/JVI.75.21.10118-10131.2001 11581380 PMC114586

[B19] Chi XS Hu A Bolar Al-Rimawi W Zhao P Tam JS Rappaport R Cheng SM Detection and Characterization of New Influenza B Virus Variants in 2002 J Clin Microbiol 2005 43 2345 2349 10.1128/JCM.43.5.2345-2349.2005 15872264 PMC1153786

